# Naturally presented HLA class I–restricted epitopes from the neurotrophic factor S100-β are targets of the autoimmune response in type 1 diabetes

**DOI:** 10.1096/fj.201802270R

**Published:** 2019-02-28

**Authors:** Cristina Calviño-Sampedro, Iria Gomez-Tourino, Oscar J. Cordero, Pedro A. Reche, Marta Gómez-Perosanz, Jose Luis Sánchez-Trincado, Miguel Ángel Rodríguez, Aurelio M. Sueiro, Juan E. Viñuela, Rubén Varela Calviño

**Affiliations:** * Departamento de Bioquímica y Biología Molecular, Facultad de Farmacia, Universidade de Santiago de Compostela, Santiago de Compostela, Spain;; † Departamento de Inmunología, Facultad de Medicina, Universidad Complutense de Madrid, Madrid, Spain;; ‡ Departamento de Biología Funcional, Centro de Investigación en Bioloxía (CIBUS), Universidade de Santiago de Compostela, Santiago de Compostela, Spain;; § Servicio de Endocrinología y Nutrición, Complejo Hospitalario Universitario de Santiago de Compostela (CHUS)–Hospital de Conxo, Santiago de Compostela, Spain; and; ¶ Servicio de Inmunología, Hospital Clínico Universitario, Complejo Hospitalario Universitario de Santiago de Compostela (CHUS), Santiago de Compostela, Spain

**Keywords:** cytotoxic lymphocytes, autoantigen, S100β peptide epitopes, immunotherapy, peri-insular Schwann cells

## Abstract

Type 1 diabetes (T1D) results from the destruction of pancreatic β-cells by the immune system, and CD8^+^ T lymphocytes are critical actors in this autoimmune response. Pancreatic islets are surrounded by a mesh of nervous cells, the peri-insular Schwann cells, which are also targeted by autoreactive T lymphocytes and express specific antigens, such as the neurotrophic factor S100-β. Previous work has shown increased proliferative responses to whole S100-β in both human T1D patients and the nonobese diabetic (NOD) mouse model. We describe for the first time naturally processed and presented epitopes (NPPEs) presented by class I human leukocyte antigen–A*02:01 (A2.1) molecules derived from S100-β. These NPPEs triggered IFN-γ responses more frequently in both newly diagnosed and long-term T1D patients compared with healthy donors. Furthermore, the same NPPEs are recognized during the autoimmune response leading to diabetes in A2.1-transgenic NOD mice as early as 4 wk of age. Interestingly, when these NPPEs are used to prevent diabetes in this animal model, an acceleration of the disease is observed together with an exacerbation in insulitis and an increase in S100-β–specific cytotoxicity in vaccinated animals. Whether these can be used in diabetes prevention needs to be carefully evaluated in animal models before use in future clinical assays.—Calviño-Sampedro, C., Gomez-Tourino, I., Cordero, O. J., Reche, P. A., Gómez-Perosanz, M., Sánchez-Trincado, J. L., Rodríguez, M. Á., Sueiro, A. M., Viñuela, J. E., Calviño, R. V. Naturally presented HLA class I–restricted epitopes from the neurotrophic factor S100-β are targets of the autoimmune response in type 1 diabetes.

One of the hallmarks of type 1 diabetes (T1D) in both human patients and nonobese diabetic (NOD) mice is the destruction of the insulin-producing β-cells in the pancreatic islets. The autoimmune origin of this attack comes from several lines of evidence, including the detection of autoantibodies years before clinical disease onset (1) and the detection of T lymphocytes specific for the very same antigens (2–5).

During this process, pancreatic islets are infiltrated by different immune cell types, including CD4^+^ and CD8^+^ T lymphocytes (4, 6, 7). However, CD8^+^ lymphocytes are critical for diabetes development. First, neither insulitis nor diabetes develops in NOD mice lacking major histocompatibility complex (MHC) class I molecules and CD8^+^ T cells (8). Second, in human T1D, there is a linkage to susceptibility to particular human leukocyte antigen (HLA) class I molecules, such as A*2401 and A*0201, and also to protection, such as A*01 and A*1101 (9, 10). Finally, in human T1D patients, CD8^+^ T lymphocytes are the predominant cell subset seen in insulitic lesions linked to a hyperexpression of HLA class I molecules (2, 11, 12).

Several targets of this inflammatory response have been identified, among which preproinsulin, glutamic acid decarboxylase 65 (GAD_65_), and insulinoma-associated protein 2 (IA-2)–β have been repeatedly cited in the literature (13). Recent results in both the NOD mice and human T1D patients have led to the identification of other important targets for those autoreactive T lymphocytes, such as zinc transporter 8 or islet-specific glucose-6-phosphatase–related protein (14, 15).

However, during diabetes development, peri-insular Schwann (pSC) cells are also targeted by autoreactive lymphocytes (16). pSC cells organize a network, forming a mesh surrounding the islets (16, 17). In NOD mice, lymphocytic infiltration leads to the breakdown of the pSC network and the development of insulitis (17, 18). pSC cells express specific antigens, such as glial fibrillary acidic protein (GFAP) and the neurothrophic factor S100-β. Those antigens have been shown to be targeted by autoantibodies (19, 20) and T lymphocyte responses (16, 21–25). Lymphocyte proliferation against GFAP and S100-β antigens has been demonstrated in NOD mice and human T1D patients (16, 22). In the case of GFAP, I-A^g7^ and *K*^d^ peptide epitopes have been described in NOD mice (21), and HLA-A2.1–restricted peptide epitopes have been described in humans (24). Recently, naturally processed and presented epitopes (NPPEs) derived from S100-β, restricted by the HLA-DRB1*04:01 (HLA-DR4) class II molecule, and targeted by the autoimmune response in T1D patients have been identified (23). In NOD mice, peptide epitopes derived from S100-β and recognized by CD4^+^ T lymphocytes have also been described (25); interestingly, dominant S100-β–derived epitopes in NOD mice lie within the same regions as those targeted by human T responses.

Immunotherapies employing either whole GFAP or S100-β protect NOD mice from diabetes development (16). Moreover, immunotherapy with class II I-A^g7^– and class I K^d^–restricted peptide epitopes derived from GFAP protect NOD mice from diabetes development (21). These data indicate that immunotherapy with pSC-derived antigens could prevent the development of clinical symptoms.

Despite these results, no S100-β peptide epitopes targeted by CD8^+^ cytotoxic T lymphocytes (CTLs) in T1D patients have been identified, nor has their potential effectiveness in preventing diabetes development been reported. In the present study, we have identified several NPPEs derived from S100-β and presented by the class I HLA molecule A*02:01 (A2.1). We hypothesized that responses against these peptide epitopes might be present in both human T1D patients and A2.1-transgenic NOD mice. Our results support this hypothesis: higher responses against some S100-β–derived NPPEs can be detected more frequently in both newly diagnosed (ND) and long-term T1D patients. Furthermore, T1D patients respond more frequently and with higher intensity to more than 1 epitope. Similar responses can be detected in A2.1-transgenic NOD mice as early as 4–5 wk of age. When these peptide epitopes were used for immunotherapy, an apparent acceleration of disease development was observed together with a worsening in the insulitic lesions and increased cytotoxicity against S100-β–positive targets by CTLs. Our results highlight the need for careful evaluation of different administration routes and dosage protocols using this antigen to ameliorate the autoimmune response in T1D.

## MATERIALS AND METHODS

### Cloning of S100-β and generation of an S100-β–expressing surrogate antigen-presenting cell

S100-β cDNA (GenBank accession no. BC001766.1) was obtained from SourceBioscience (Nottingham, United Kingdom) and subcloned into the *BamHI* site of the pcDNA3.1/Zeo(+) vector (Thermo Fisher Scientific, Waltham, MA, USA).

A surrogate antigen-presenting cell was generated by transfection of an HLA-A2.1–expressing K562 cell line (K562/A2.1) with S100-β/pcDNA3.1-Zeo using Lipofectamine (Thermo Fisher Scientific) following the manufacturer’s instructions. Clones expressing high levels of S100-β were selected using Geneticin and Zeocin (Thermo Fisher Scientific).

Expression of both A2.1 and S100-β was verified by flow cytometry and immunofluorescence. For flow cytometry, cells were surface stained with an anti-human HLA-A2.1–FITC mAb (BB7.2 clone) (BD Biosciences, San Jose, CA). After washing, cells were fixed and permeabilized (Cytofix/Cytoperm; BD Biosciences) and stained intracellularly with a mouse anti–S100-β mAb (Abcam, Cambridge, MA, USA) followed by an anti-mouse IgG phycoerythrin-labeled goat pAb (Abcam) and analyzed using a BD FACScalibur flow cytometer (BD Biosciences).

For immunofluorescence analysis, cells were fixed onto poly-l-Lysine–coated coverslips; stained with either the BB7.2-FITC antibody (surface) or, after fixation/permeabilization, the S100-β–FITC antibody (Abcam) (intracellular) and DAPI; and imaged in an Olympus BX51 fluorescence microscope (Olympus, Tokyo, Japan).

### Identification of HLA-A2.1–restricted NPPEs derived from S100-β

Peptides bound to A2.1 in the cell surface of either K562/A2.1 or K562/A2.1–S100-β were eluted by a brief incubation in acid citrate buffer (pH 3.3) and sequentially enriched using a 3 kDa cutoff Amicon Ultra Filter (MilliporeSigma, Burlington, MA, USA) and a Discovery DSC-18 trifunctional C18 silica resin column (MilliporeSigma). Peptide fractionation was done using a 150 mm × 2.1 mm BioBasic 18 column (Thermo Fisher Scientific), and peptide-containing fractions were stored at −80°C until analysis by mass spectrometry (MS).

MS was carried out in the Spectrometry Service [Instituto de Investigaciones Sanitarias (IDIS), Santiago de Compostela] on a matrix-assisted laser desorption/ionisation–time of flight mass spectrometry (MALDI-TOF) MS Analyzer (Thermo Fisher Scientific). The analysis was carried out with the 4000 Series Explorer software v.3.5 (Thermo Fisher Scientific) and Mascot v.2.1 (Matrix Science, Boston, MA, USA) to search against a National Center for Biotechnology Information nonredundant (NCBInr) protein database or in an S100-β–specific database. Unique *m/z* values were identified using Findpept (*http://web.expasy.org/findpept/*) to select those that could be derived from S100-β. Potential unique peptide epitopes of 8–10 aa long were chosen.

### *In vitro* proteasome digestion and *in silico* proteasomal cleavage analysis of purified human S100-β

Purified human S100-β (23) was incubated with purified 20S proteasome (Enzo Life Sciences, Farmingdale, NY, USA) (molar ratio 250:1) in digestion buffer [30 mM Tris-HCl (pH 8.0), 10 mM NaCl, 2 mM MgCl_2_, 1 mM DTT, 0.01% sodium dodecyl sulfate) for 16 h at 37°C. As a control, the same digestion was set up with either no proteasome or with acetic acid (1%)–inactivated proteasome. Generated peptides were purified and concentrated using a cationic resin (ZipTip with strong cation exchange; MilliporeSigma) following the manufacturer’s instructions. Retained peptides were eluted from the resin and analyzed by MS.

The S100-β human protein sequence was analyzed *in silico* for potential proteasome and immunoproteasome cleavage sites using several published algorithms (26, 27).

### Peptides

S100-β–derived NPPEs S100_10–18_ (ALIDVFHQY) and S100_20–28_ (GREGDKHKL) were synthesized by ChinaPeptide (Hangzhou, China) to >90% purity. A stock of 100 mg/ml in DMSO was prepared for each peptide and stored at −20°C until use.

### HLA stabilization assay

Binding of the S100-β NPPE candidates was examined by a conventional HLA stabilization assay as previously described (28). Briefly, T2 cells were washed twice with serum-free AIM V medium (Thermo Fisher Scientific) and incubated in medium containing β2-microglobulin (MilliporeSigma) and each of the test peptides at various concentrations (100–0.78 μM). Surface A2.1 expression was determined using the BB7.2 antibody in a FACSCalibur flow cytometer.

### Human donors

In total, 35 subjects were studied: 18 nondiabetic healthy donors (HDs) without family history of disease and 7 ND (<1 yr) and 10 long-standing (LS) (>1 yr) T1D patients. Blood was drawn with the informed consent of all subjects and appropriate permission was obtained from the Institutional Ethics Committee (Comité Ético de Investigación Clínica de Galicia, CEIC). T1D patients were enrolled from those attending the diabetic clinic at the Endocrinology Service (Hospital de Conxo, Santiago de Compostela, Spain). Healthy nondiabetic donors were recruited from laboratory and hospital staff during the same period. HLA-DR4 and HLA-A2.1 positivity was determined by PCR as previously described by Bunce (29). GAD_65_ and IA-2 autoantibodies were determined by ELISA (Palex Medical, Barcelona, Spain).

### Mice

Transgenic NOD.B6-Tg(HLA-A2.1)1Enge/DvsJ mice were purchased from The Jackson Laboratory (Bar Harbor, ME, USA) and maintained in the specific-pathogen–free facilities at the Molecular Medicine and Chronic Diseases Research Centre (Santiago de Compostela). Glucosuria was determined weekly from 8 wk of age onward using Medi-Test Glucose Strips (Macherey-Nagel, Düren, Germany). Diabetes was diagnosed when glucose levels were ≥500 mg/dl (27.8 mM) in 2 consecutive measures. All experimental procedures were approved by the corresponding ethics committee. Splenocytes were stained with the BB7.2 antibody to confirm A2.1 expression.

To obtain single-cell suspensions, spleens were passed through a 70-μm pore size filter (Corning cell strainer; Corning, Corning, NY, USA) and centrifuged over a Ficoll gradient.

### S100-β NPPE immunotherapy

An immunotherapy protocol was performed as previously described by Han *et al.* (30). Briefly, female mice were immunized intraperitoneally with a 1:1 mixture of S100_10–18_ and S100_20–28_ in saline. Immunizations started at 4 wk of age and were performed every 2 wk until 8 wk of age or 3 wk thereafter. Mice were followed until they became diabetic or reached 30 wk of age. High (100 μg/peptide) and low (10 μg/peptide) peptide doses were evaluated. As a control, female mice were inoculated with saline containing DMSO (0.2%).

### Insulitis and immunohistochemistry

Bouin-fixed pancreases were included in paraffin, and 3 parallel series with 16 μm thick sections were obtained. Sections were stained with hematoxylin-eosin, and between 30 and 50 islets per mouse were scored in a blinded fashion. Quantification of insulitis with age was done using 5–6 animals per age group. Insulitis score in treated animals was determined after diabetes diagnosis or at 30 wk of age. Each islet was scored according to the scale indicated in the supplemental data.

For immunohistochemistry, pancreases were fixed in 4% paraformaldehyde. Sections were pretreated with Proteinase K (10 mg/ml) for 5 min at room temperature. Sections were rinsed in 0.05 M Tris-buffered saline (TBS) (pH 7.4) for 5 min and treated with 10% H_2_O_2_ in TBS (30 min, room temperature). After rinsing in 0.05 M TBS (pH 7.4) for 5 min, sections were incubated overnight at room temperature with an anti-GFAP mouse mAb (clone 2A5; Abcam). Sections were rinsed 3 times in TBS (10 min each) and incubated with an anti-mouse horseradish peroxidase–coupled secondary antibody (1 h, room temperature). Sections were developed with 0.25 mg/ml diaminobenzidine tetrahydrochloride (MilliporeSigma) in TBS (pH 7.4) and 0.00075% H_2_O_2_ and counterstained with hematoxylin-eosin. All dilutions were made with TBS containing 15% normal goat serum (MilliporeSigma) and 0.2% Triton X-100 (MilliporeSigma). All incubations were carried out in a humid chamber. Finally, the sections were dehydrated and mounted.

### Enzyme-linked immunospot assays

IFN-γ enzyme-linked immunospot (ELISPOT) assays (U-CyTech Biosciences, Utrecht, The Netherlands) were used to detect S100-β–specific responses according to the manufacturer’s instructions. Mouse splenocytes were used in all assays, and an indirect ELISPOT assay was performed with a 6-h prestimulation step. When human responses were analyzed, a direct ELISPOT assay was performed (3 × 10^5^ peripheral blood mononuclear cells (PBMCs)/well in triplicates per antigen) with a 24-h stimulation period with the corresponding peptide/antigen.

Peptides were added to a final concentration of 10 μM. Both culture medium alone and DMSO (0.5%) were used as negative controls. CEF Peptide Pool (Mabtech AB, Nacka, Sweden) or concanavalin A (25 ng/ml) were used as positive controls in human and mouse ELISPOTs, respectively.

Data are expressed as a stimulation index (SI) (SI = total spots with antigen/total spots with DMSO). In human ELISPOTs, a response was considered positive when the SI was above the threshold determined using receiver-operating characteristic (ROC) curves. In mouse ELISPOTs, a response was considered positive when the SI was >2.

### Cytotoxicity assays

Lysis of peptide-loaded targets was done as previously indicated by Neri *et al.* (31). Briefly, splenocytes from young nondiabetic female mice were used as targets, whereas splenocytes from either saline- (*n* = 5) or S100-β– (*n* = 13) vaccinated mice were used as effectors. Target cells were incubated 2 h with S100-β NPPEs (both at 10 nM) or DMSO and stained with calcein (15 mM) (MilliporeSigma). Cells were washed, and 1 × 10^4^ cells were plated in U-bottom, 96-well plates. Effector cells were added in quadruplicates at different effector:target ratios (1:1 to 40:1). Maximum lysis was obtained by adding 2% Triton X-100 to target cells, and spontaneous lysis was obtained by adding culture medium to target cells. After 4 h of incubation, fluorescence (530 nM) in culture medium was determined in a Synergy H1M plate reader (BioTek Instruments, Winooski, VT, USA). The percentage of cytotoxicity was calculated as (MF_sample_ − MF_spontaneous_)/(MF_maximum_ − MF_spontaneous_) × 100, where MF is mean fluorescence.

### Statistical analysis

Differences in the percentage of highly infiltrated islets and the frequency of positive responses among different groups were analyzed using Fisher’s exact test. Comparison of median responses in ELISPOT assays between groups was done using the Mann-Whitney *U* test or the Kruskal-Wallis test for multiple comparisons. Survival was analyzed using the log-rank Mantel-Cox test. A value of *P* ≤ 0.05 was considered significant. All statistical analysis was done using SPSS v.24 (IBM SPSS, Chicago, IL, USA) and Prism (GraphPad, La Jolla, CA, USA).

## RESULTS

### Generation of surrogate antigen-presenting cells for the identification of A2.1-restricted S100-β–derived NPPEs

Several clones were obtained from the original K562-A2.1/S100-β cell line, and 4 were selected based on their expression of both A2.1 and S100-β (Supplemental Fig. S1*A*, *B*), confirming the generation of a surrogate antigen-presenting cell.

No significant differences were noted between chromatograms from K562/A2.1 and K562/A2.1–S100-β–eluted peptides (Supplemental Fig. S1*C*). In total, 44 peptide-containing fractions were analyzed by MS, identifying 7298 and 7027 *m/z* masses from K562/A2.1 and K562/A2.1–S100-β cells, respectively. Up to 3403 *m/z* could be derived from S100-β, allowing a 1 Da error, with just 2035 *m/z* remaining when only peptides between 8 and 11 aa long were kept. These *m/z* masses were compared with the equivalent fraction derived from K562/A2.1 cells as well as the immediately preceding and posterior fractions looking for unique masses. This analysis led to the identification of 4 unique candidate masses (**Table 1**). Most predictions for each experimental *m/z* correspond either to overlapping peptides or peptides lying within the same short region of S100-β (Table 1).

**TABLE 1:** NPPEs derived from S100-β and presented by the A2.1 molecule

Experimental *m/z*	Theoretical *m/z*	∆Da	Sequence	Position
935.26	934.491	−0.934	VDKVMTEL	54–61
	935.359	−0.067	ETLDNDGDG	59–67
	935.359	−0.067	TLDNDGDGE	60–68
	936.398	0.971	VMETLDND	57–64
974.290	973.474	−0.816	INNELSHF	37–44
	973.474	−0.816	NNELSHFL	38–45
	973.520	−0.769	EIKEQEVV	47–54
	973.640	−0.649	KLKKSELK	27–34
	974.356	0.065	GECDFQEF	67–74
	974.515	0.225	KEQEWDK	49–56
	974.588	0.298	LKKSELKE	28–35
	974.588	0.298	KKSELKEL	29–36
1039.565	1039.480	−0.084	QYSGREGDK	17–25
	1039.564	0.000	GREGDKHKL	20–28
1105.506	1105.396	−0.109	GECDFQEFM	67–75
	1105.568	0.061	ALIDVFHQY	10–18

∆Da, difference between experimental and theoretical *m/z* (in Da); Experimental *m/z*, unique *m/z* present only in the peptide mix derived from K562/A2.1–S100-β cells; Position, amino acid position in the S100-β protein of the predicted peptide; Sequence, amino acid sequence of the predicted peptide; Theoretical *m/z*, *m/z* predicted by Findpept.

### Proteasome cleavage and A2.1 binding affinity of candidate S100-β NPPEs

**Figure 1:**
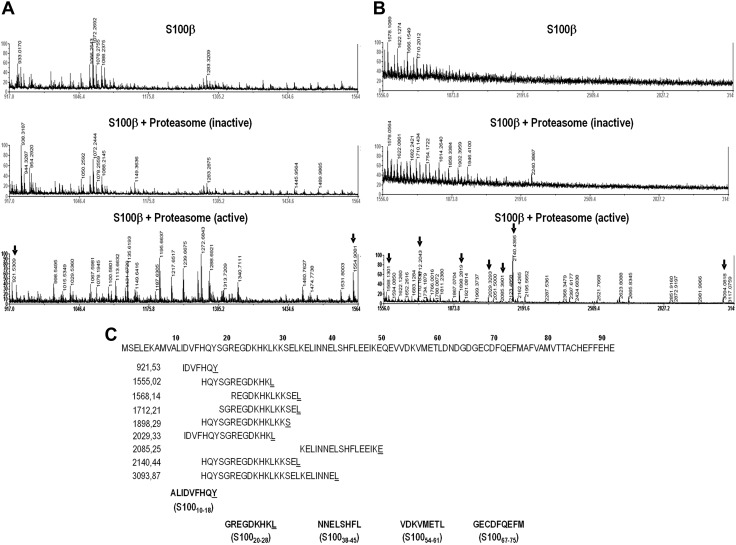
MS analysis of *in vitro* proteasome digestion of purified S100-β. *A*, *B*) Spectra corresponding to *m/z* range of 917–1564 (*A*) and 1556–3145 (*B*) are shown. Data for S100-β incubated with no proteasome (top panels), inactive proteasome (middle panels), or active proteasome (bottom panels) are shown. Unique *m/z* present only in samples of S100-β incubated with active proteasome were subsequently identified by tandem MS and analyzed by Mascot (arrows). *C*) Sequences of the 9 most intense fragments generated by *in vitro* digestion of S100-β with purified 20S proteasome are aligned with the amino acid sequence of the antigen. The *m/z* for each fragment is shown on the left. The carboxy-terminal amino acid is underlined. The sequences of potential peptide epitopes eluted from A2.1 are shown at the bottom (carboxy-terminal amino acid is underlined only if the fragment has been generated by the proteasome and identified by MS). The carboxy-terminal ends of peptides S100_10–18_ (ALIDVFHQY) and S100_20–28_ (GREGDKHKL) are generated by the proteasome, making them potential class I–restricted peptide epitopes.

From all the sequences shown in Table 1, potential proteasome cleavage sites are predicted by several algorithms for 5 peptide sequences (10–18 ALIDVFHQY; 20–28 GREGDKHKL; 38–45 NNELSHFL; 54–61 VDKVMETL; 67–75 GECFQEFM). We next determined the *in vitro* proteasome cleavage pattern of S100-β by digestion with purified 20S proteasome. The use of active proteasome generates multiple unique fragments (**Fig. 1*A, B***, bottom panels), which were not present when S100-β was incubated alone (Fig. 1*A, B*, top panels) or with inactivated proteasome (Fig. 1*A, B*, middle panels). Unique *m/z* present in samples of S100-β incubated with active proteasome were analyzed by tandem MS and Mascot, and 9 of them were identified as being derived from S100-β (Fig. 1*A, B*, bottom panels, arrows), with many of these corresponding to its amino-terminal end (Fig. 1*C*). Comparison of proteasome-generated peptides with A2.1-eluted peptide MS data (Table 1) indicates that the carboxy-terminal end of peptides S100_10–18_ (ALIDVFHQY) and S100_20–28_ (GREGDKHKL) is generated by the proteasome, making these sequences potential HLA class I–restricted epitopes. MS data and proteasome cleavage were the 2 main criteria to select the NPPEs to be evaluated in the ELISPOT assays.

Of the 5 peptides with the potential proteasome cleavage sites indicated above, only peptide S100_10–18_ (ALIDVFNQY) demonstrated some weak binding affinity for A2.1, whereas for the other 4 S100-β peptides, no binding affinity could be demonstrated (Supplemental Fig. S2*A*). Interestingly, several algorithms (32–35) indicate that S100_10–18_ is one of the peptides with the highest binding affinity for A2.1 from all of the potential 9-mer peptides derived from S100-β (Supplemental Fig. S2*B*). In fact, when combining predictions for processing by the proteasome, transporter associated with antigen processing binding, and A2.1 binding affinity (36), S100_10–18_ is once again the peptide showing the highest combined prediction score, followed by S100_67–75_ and S100_20–28_ (Supplemental Fig. S2*B*). All of these data combined suggest that peptide epitopes S100_10–18_ (ALIDVFHQY) and S100_20–28_ (GREGDKHKL) could constitute potential candidates to be recognized by autoreactive CD8^+^ T lymphocytes in T1D patients.

### T1D patients show specific T-cell responses against A2.1-restricted S100-β–derived NPPEs

**Figure 2:**
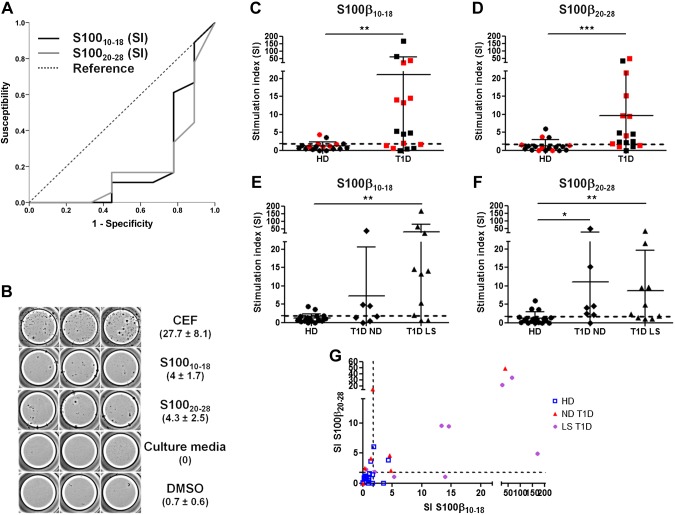
IFN-γ ELISPOT analysis of responses against S100_10–18_ and S100_20–28_ peptide epitopes. *A*) ROC curves were performed to discriminate optimal cutoff values between responders and nonresponders, giving an SI cutoff of 1.85 and 1.75 for S100_10–18_ and S100_20–28_, respectively. An SI ≥2 was used as a cutoff. *B*) A representative response from a T1D patient is shown (in parentheses, spot mean number ± sd of triplicate wells) for S100_10–18_ and S100_20–28_. A peptide mixture containing known peptide epitopes derived from viral proteins (CEF) was included as a positive control. Spontaneous responses were determined by PBMC culture in medium alone (culture medium) or medium containing 0.5% DMSO. *C–F*) IFN-γ secretion in response to S100_10–18_ (*C, E*) and S100_20–28_ (*D, F*) by PBMCs from HDs, all T1D patients (T1D), ND T1D patients (T1D-ND), and LS T1D patients (T1D-LS). Black symbols: HLA-A2.1–negative subjects; red symbols: HLA-A2.1–positive subjects. Kruskal-Wallis test with Dunn’s *post hoc* test (*C*, *D*). *P < 0.05, **P < 0.01, ***P < 0.001. *G*) Responses against both S100_10–18_ and S100_20–28_ were more frequently detected among T1D patients (ND T1D: 57.1%; LS T1D: 60%) compared with HDs (11.1%). Two-tailed Fisher’s exact test, *P* = 0.0045. There is also a positive correlation between the responses to both peptides (*r* = 0.66, Spearman’s correlation, *P* < 0.0001). The dashed line (*C*–*G*) represents the threshold.

We investigated whether lymphocyte responses against S100_10–18_ and S100_20–28_ could be detected in T1D patients by IFN-γ ELISPOT (see **Table 2** for demographic data). ROC analysis gives an optimal SI cutoff of 1.85 and 1.75 for S100_10–18_ and S100_20–28_, respectively (**Fig. 2*A***); therefore, an SI ≥2 was chosen to classify a response as positive.

**TABLE 2:** HD and T1D patient demographics

Variable	HD (*n* = 19)	ND (*n* = 7)	LS (*n* = 11)
Age [mean ± sd (yr)]	21 ± 1.1	34.6 ± 11.8	43.4 ± 15.5
Sex (M/F)	5/14	3/4	5/6
Time since diagnosis [mean ± sd (mo)]	N/A	2.6 ± 1.2	53.1 ± 75.2
Time since diagnosis [range (mo)]	N/A	1.0–4.5	10–267
GADA positive (%)	N/A	71.4	72.7
IA-2A positive (%)	N/A	42.9	36.4
HLA-DR4 positive (%)	15.8	0	50
HLA-A2.1 positive (%)	31.6	40	58.4

F, female; IA-2A, islet antigen 2 autoantibodies; GADA, GAD_65_ autoantibodies; M, male; N/A, not applicable.

Clear responses against at least 1 of the 2 S100-β peptides could be detected (Fig. 2*B*), and these responses were significantly more frequent among T1D patients than HDs for both S100-β peptide epitopes [S100_10–18_: 64.7% T1D (11/17) *vs.* 11.1% HD (2/18). *P* = 0.0016; S100_20–28_: 70.6% T1D (12/17) *vs.* 16.7% HD (3/18). *P* = 0.002. Two-tailed Fisher’s exact test]. Positive responses to S100_10–18_ could be detected in both HLA-A2.1–positive (*n* = 6; Fig. 2*C*, red squares) and HLA-A2.1–negative (*n* = 5; Fig. 2*C*, black squares) T1D patients. A similar result is seen for S100_20–28_ in HLA-A2.1–positive (*n* = 7; Fig. 2*D*, red squares) and HLA-A2.1–negative (*n* = 6; Fig. 2*D*, black squares) T1D patients. Considering only HLA-A2.1–positive subjects, responses in A2.1+ T1D patients are still significantly higher compared with HD A2.1+ subjects for S100_20–28_ (*P* = 0.04; 2-tailed Fisher’s exact test) but not for S100_10–18_ (*P* = 0.30; 2-tailed Fisher’s exact test).

The magnitude of the responses (median SIs) is also higher among T1D patients [S100_10–18_: 4.8 (T1D) *vs.* 1.0 (HD). *P* = 0.004. S100_20–28_: 4.1 (T1D) *vs.* 0.9 (HD). *P* = 0.0006. Mann-Whitney *U* test] (Fig. 2*C, D*). Considering only HLA-A2.1–positive subjects, responses in A2.1+ T1D patients are still significantly higher compared with HD A2.1+ subjects for S100_20–28_ (*P* = 0.02; Mann-Whitney *U* test) and almost significant for S100_10–18_ (*P* = 0.09; Mann-Whitney *U* test).

We next decided to compare the responses between ND and LS T1D patients. In the case of S100_10–18_, the frequency of positive responses among ND (4/7; 57.1%) and LS (8/10; 80.8%) T1D patients was higher compared with that seen in HDs (2/10; 20.0%), reaching statistical significance only for LS T1D patients (*P* = 0.002; 2-tailed Fisher’s exact test) (Fig. 2*E*). In the case of S100_20–28_, frequency of positive responses among ND (6/7; 85.7%) and LS T1D patients (6/10; 60.0%) was again higher compared with that seen in HD (2/10; 20%), reaching statistical significance only for ND T1D patients (*P* = 0.015; 2-tailed Fisher’s exact test) (Fig. 2*F*).

Furthermore, the magnitude of the responses (median SIs) against S100_10–18_ were significantly higher in LS T1D patients (13.7) *vs.* HD (1.0) (Fig. 2*E*) (*P* = 0.0024; Kruskal-Wallis, Dunn’s *post hoc* test). In the case of S100_20–28_, median responses in ND (4.1) and LS (3.6) T1D patients were significantly higher compared with HD (0.9) (Fig. 2*F*) (*P* = 0.01 and *P* = 0.001, respectively; Kruskal-Wallis, Dunn’s *post hoc* test).

Interestingly, dual positivity (*i.e.*, response to both peptides) is more frequently detected in T1D patients (9/17; 52.9%) compared with HDs (1/18; 5.5%) (*P* = 0.0027; 2-tailed Fisher’s exact test) (Fig. 2*G*).

In summary, all these data indicate that proinflammatory lymphocyte responses against the 2 novel S100-β NPPEs are more frequent in T1D patients, both ND and LS.

### Immune responses against S100-β–derived NPPEs in the A2.1-transgenic NOD mice

We examined whether similar responses against the S100_10–18_ and S100_20–28_ peptide epitopes could be detected in the A2.1-transgenic NOD mouse preclinical model (37). Diabetes development kinetics and insulitis in both males and females in our colony are shown in Supplemental Fig. S3.

**Figure 3:**
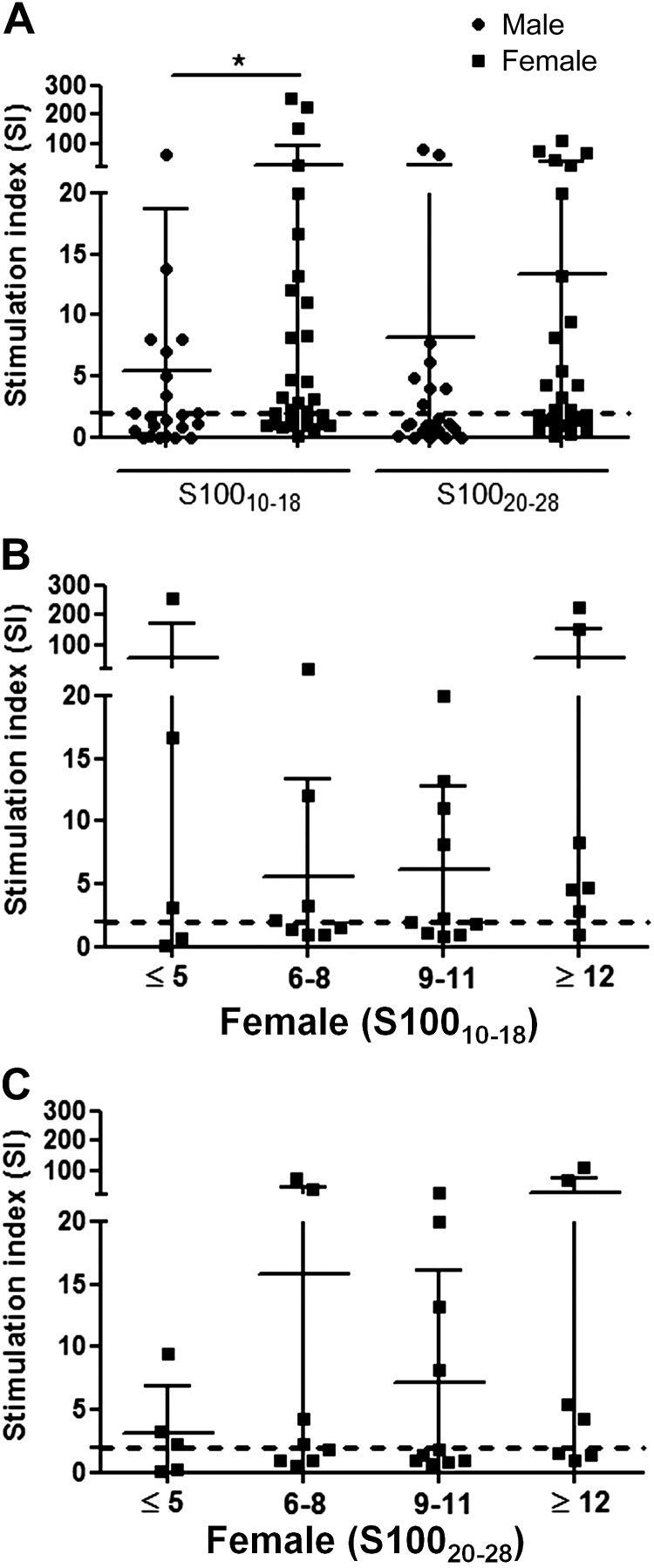
Spontaneous autoimmune responses against S100_10-18_ and S100_20-28_ in A2.1-transgenic NOD mice. *A*) Spontaneous autoimmune responses against S100_10–18_ and S100_20–28_ were determined in splenocytes by IFN-γ ELISPOT in male (*n* = 22; 10.32 ± 4.76 wk of age, mean ± sd) and female (*n* = 31; 9.83 ± 3.97, mean ± sd) A2.1-transgenic NOD mice. Data shown are the SIs (*y* axis) calculated as the ratio between the number of spots with peptide and the number of spots in culture medium only. SIs ≥2 were considered positive responses. Median responses were higher in females compared with males for S100_10–18._ *B*, *C*) Mann-Whitney *U* test. *P* = 0.035. Spontaneous responses against S100_10–18_ (*B*) and S100_20–28_ (*C*) according to age were determined in female mice (*n* = 31; 5–10 animals/age group). Positive responses (SI ≥2) can be detected as early as 5 wk of age for both peptide epitopes. The dashed line represents the threshold.

Positive responses by splenocytes against S100_10–18_ and S100_20–28_ can be detected in male [8/22 (36.4%) and 8/22 (36.4%), respectively] (**Fig. 3*A***) and female mice [19/31 (61.3%) and 15/31 (48.4%), respectively] (Fig. 3*A*). There are no statistical differences in the percentage of responses between male and female mice (*P* = 0.09, *P* = 0.41; 2-tailed Fisher’s exact test). However, the magnitude of the responses (median SIs) is significantly higher among females (2.6) when compared with males (1.5) for S100_10–18_ (*P* = 0.035; Mann-Whitney *U* test) (Fig. 3*A*).

When those responses were examined according to age in female mice, responses to both S100-β–derived NPPEs can be detected as early as 4–5 wk of age (Fig. 3*B, C*).

These data confirm that S100-β is a target of the autoimmune response in T1D and that epitopes S100_10–18_ and S100_20–28_ are targeted by T lymphocytes.

### Immunotherapy of A2.1-transgenic NOD mice with S100-β–derived peptides

**Figure 4:**
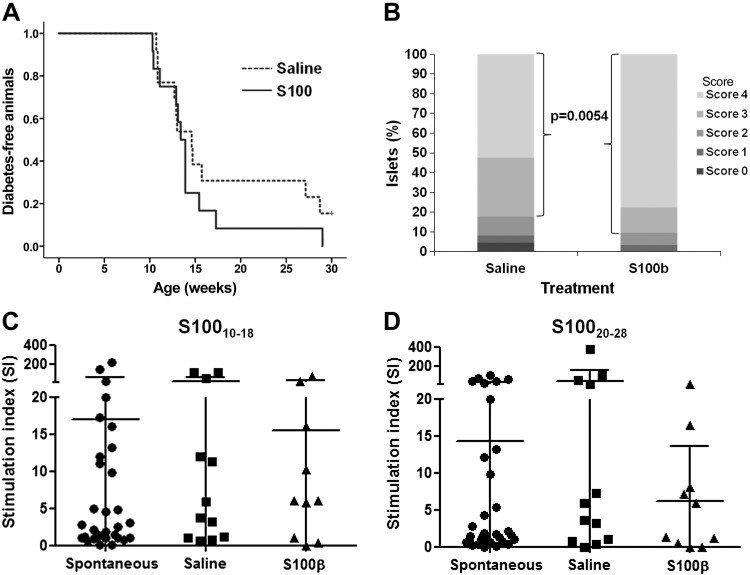
T1D development in A2.1-transgenic NOD mice vaccinated intraperitoneally with S100_10–18_ and S100_20–28_. *A*) Animals were vaccinated with 100 μg each of S100_10–18_ and S100_20–28_ per dose (*n* = 10; continuous line) starting at 4 wk of age and then every 2–3 wk until they developed diabetes or reached 30 wk of age. Animals inoculated with saline (*n* = 12; dotted line) were used as controls. Vaccination with S100_10–18_ and S100_20–28_ does not seem to significantly modify either the incidence or the kinetics of diabetes development in A2.1-transgenic NOD mice. Log-rank, Mantel-Cox, *P* = 0.29. *B*) Insulitis in A2.1-transgenic NOD mice vaccinated with S100-β–derived NPPEs or inoculated with saline. The percentage of islets heavily infiltrated (scores 3 and 4) is significantly higher in S100-β–vaccinated animals compared with those inoculated with saline. Fisher’s exact test; *P* = 0.0054. *C*, *D*) IFN-γ secretion in response to S100_10–18_ (*C*) and S100_20–28_ (*D*) in A2.1-transgenic NOD mice vaccinated with the S100-β–derived NPPEs (S100-β), saline (saline), or nonmanipulated (spontaneous). In all cases, splenocytes were harvested at disease diagnosis or at 30 wk of age. Neither frequency of positive responses (Fisher’s exact test, *P* > 0.05) nor median SI responses (Kruskal-Wallis test, *P* = 0.4) were significantly different in S100-β–immunized mice compared with spontaneous and saline-inoculated mice.

We next evaluated the capacity of these NPPEs to prevent disease development by periodic immunization of young female mice. No significant reduction in disease development frequency is seen compared with saline-inoculated mice (**Fig. 4*A***) (100% *vs.* 84.6%; *P* = 1.0; 2-tailed Fisher’s exact test). In fact, S100-β vaccination seems to slightly accelerate T1D development, although this kinetic does not reach statistical significance (Fig. 4*A*) (*P* = 0.290; log-rank, Mantel-Cox). However, vaccination does have an effect on the cellular autoimmune response, as the number of highly infiltrated islets is significantly higher in S100-β–vaccinated animals (Fig. 4*B*) (*P* = 0.0054; Fisher’s exact test). Moreover, numerous GFAP-immunoreactive cells are observed around the islets of young nondiabetic control female mice (Supplemental Fig. S4*A*–*D*) compared with diabetic S100-β–immunized female mice, in which these cells are scarce and show a faint GFAP immunoreactivity (Supplemental Fig. S4*E*–*H*, see arrows in panels *F* and *H*). Some immunoreactive intra-islet cells are also seen in the pancreatic islets of control animals (Supplemental Fig. S4, arrowheads in panels B and D), which are absent in the immunized animals. These results show a decrease in the number of pSC cells in the S100-β–immunized animals compared with young nondiabetic insulitis-free female mice.

Despite this increase in islet infiltration severity, positive responses against S100_10–18_ (70%, 7/10) (Fig. 4*C*, S100-β) or S100_20–28_ (50%, 5/10) (Fig. 4*D*, S100-β) are not significantly higher than that observed in nonmanipulated animals (S100_10–18_: 53.1%, 17/32; S100_20–28_: 48.4%, 15/31) (Fig. 4*C, D*, spontaneous) or inoculated with saline (S100_10–18_: 66.7%, 8/12; S100_20–28_: 66.7%, 8/12) (Fig. 4*C, D*, saline) (*P* > 0.05; Fisher’s exact test). Moreover, vaccination with S100-β–derived NPPEs does not significantly modify the intensity of the response measured as the median SI (*P* = 0.4; Kruskal-Wallis test).

**Figure 5:**
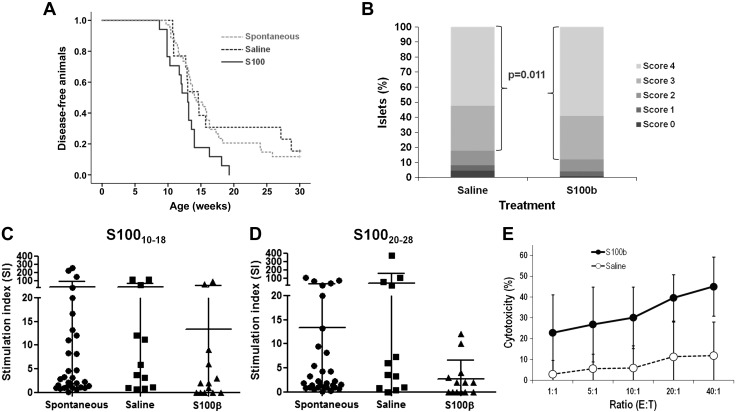
T1D diabetes development in A2.1-transgenic NOD mice vaccinated intraperitoneally with low doses of S100_10–18_ and S100_20–28_. *A*) Animals were vaccinated with 10 μg each of S100_10–18_ and S100_20–28_ per dose (*n* = 10; continuous line) starting at 4 wk of age and then every 2–3 wk until they developed diabetes or reached 30 wk of age. Animals inoculated with saline (*n* = 12; black dotted line) were used as controls. Spontaneous disease development in nonmanipulated females (*n* = 34; gray dotted line) is also shown. Vaccination with S100_10-18_ and S100_20-28_ significantly accelerates the kinetics of diabetes development in A2.1-transgenic mice compared with both saline-treated (log-rank, Mantel-Cox, *P* = 0.048) and nonmanipulated (log-rank, Mantel-Cox, *P* = 0.02) female mice. *B*) Insulitis in A2.1-transgenic NOD mice vaccinated with S100-β–derived NPPEs or inoculated with saline. The percentage of islets heavily infiltrated (scores 3 and 4) is significantly higher in S100-β–vaccinated animals compared with those inoculated with saline. Fisher’s exact test; *P* = 0.011. *C*, *D*) IFN-γ secretion in response to S100_10–18_ (*C*) and S100_20–28_ (*D*) in A2.1-transgenic NOD mice vaccinated with the S100-β–derived NPPEs (S100-β), saline (saline), or nonmanipulated (spontaneous). In all cases, splenocytes were harvested at disease diagnosis or at 30 wk of age. Neither frequency of positive responses (Fisher’s exact test, *P* > 0.05) nor median SI responses (*P* = 0.24 for S100_10–18_ and *P* = 0.097 for S100_20–28_; Kruskal-Wallis test) were significantly different in S100-β–immunized mice compared with spontaneous and saline-inoculated mice. *E*) Splenocytes from animals vaccinated with S100-β–derived NNPEs (black line) show a higher specific cytotoxicity against targets incubated with both S100-β–derived NPPEs compared with that seen in splenocytes from animals inoculated with saline (dotted line). Mann-Whitney *U* test, *P* < 0.05 for all effector:target (E:T) ratios.

To address whether the unexpected finding of high islet infiltration in S100-β–treated animals was due to the peptide doses, mice were vaccinated using doses 10 times lower. Surprisingly, disease kinetics were accelerated, reaching statistical significance when compared with both saline-treated (*P* = 0.048; log-rank, Mantel-Cox) and nonmanipulated females (Spontaneous. *P* = 0.02; log-rank, Mantel-Cox) (**Fig. 5*A***). Vaccination with the NPPEs also increased the number of highly infiltrated islets (Fig. 5*B*) (*P* = 0.011; Fisher’s exact test).

Similar to the results seen using high doses, IFN-γ responses in mice vaccinated with S100_10–18_ and S100_20–28_ [6/13 (46.1%) and 7/13 (53.8%), respectively] are not significantly higher compared with those seen in animals inoculated with saline [S100_10–18_: 66.7% (8/12); S100_20–28_: 66.7% (8/12)] or those developing T1D spontaneously [S100_10–18_: 61.3% (19/31); S100_20–28_: 48.4% (15/31)] (Fig. 5*C, D*) (*P* > 0.05; Fisher’s exact test). Moreover, vaccination with S100-β–derived NPPEs does not significantly modify the intensity of the response (*P* = 0.24 for S100_10–18_ and *P* = 0.097 for S100_20–28_; Kruskal-Wallis test). However, the immunotherapy regime seems to activate the cellular autoimmune response against S100-β because a significant increase in the cytotoxic activity against splenocytes incubated with the peptide epitopes is seen (Fig. 5*E*) (*P* < 0.05 in all effector:target ratios; Mann-Whitney *U* test), suggesting that vaccination with S100-β–derived NPPEs activates an ongoing autoimmune response rather than triggering a regulatory one.

## DISCUSSION

Islet infiltration by both CD4^+^ and CD8^+^ T cells is observed in T1D, but several studies have established an important role for the latter. In fact, the cytotoxic effector function of these cells is thought to be one of the mechanisms of β-cell destruction *in vivo* (38).

During diabetes development, pSC cells, a network of nervous cells surrounding the islet mass, are also targeted by the immune system. In NOD mice, as insulitis progresses, pSC cells are gradually eliminated (16–18). pSC cells express antigens that are either specific, such as GFAP, or shared with the β-cells, such as the neurotrophic factor S100-β (16). Interestingly, many autoantigens targeted by the immune response in T1D are expressed by different nervous and neuroendocrine tissues, such as GAD_65_, glutamic acid decarboxylase 67, islet cell antigen 512 (also referred to as IA-2), phogrin (also referred to as IA-2–β), islet cell antigen 69, or chromogranin A (39).

Our group was the first to define NPPEs derived from S100-β and restricted by the HLA-DRB1*04:01 (HLA-DR4) class II molecule. T cells from both ND and long-term T1D patients recognized the S100-β–derived NPPEs and secreted IFN-γ (23). In the present study, we defined S100_10–18_ and S100_20–28_ as new HLA-A2.1–restricted epitopes. Those epitopes were recognized more frequently by T cells from both ND and long-term T1D patients. Moreover, the frequency of dual responders (*i.e.*, those responding to both S100-β peptides) was higher in T1D patients, particularly long-term ones. Previous studies have shown that different types of autoimmune responses could be taking place in T1D patients. Differential insulitic profiles determine the extent of β-cell destruction and the age at onset (40); therefore, it remains possible that younger T1D patients than the ones shown in our study show more robust S100-β–specific cytotoxic responses, as previously shown by Banwell *et al.* for whole antigen in children using a proliferation assay (22).

S100_10–18_ lies in the same region as the HLA-DR4 peptide epitope S100_6–25_ (KAMVALIDVFHQYSGREGDK) and S100_20–28_ in the region of S100_21–36_ (REGDKHKLKKSELKEL). For other well-characterized autoantigens in T1D, MHC class I– and II–binding peptides have been identified in overlapping regions or in close proximity (21, 24, 41). Recent data in NOD mice identified dominant class II–restricted epitopes (S100_1-15_ and S100_78–92_) (25) in regions previously identified by us in human T1D (23). These data point to the fact that those regions are immunodominant in both NOD mice and human T1D patients and constitute important targets of the autoimmune response in diabetes.

Proliferative lymphocyte responses against GFAP and S100-β whole antigens have been detected in both NOD mice and human T1D patients (16, 22). Two GFAP-derived K^d^-restricted epitopes have been identified in the NOD mice. However, when evaluated for their potential in preventing diabetes, only the one with the higher MHC binding affinity was able to significantly delay T1D development (21). Our results show that responses against HLA-A2.1–restricted S100-β NPPEs can be detected as early as 4 wk of age; however, when used to prevent disease development, an acceleration was observed, more aggressive insulitis was detected, no reduction of IFN-γ secretion was seen, and an increase in cytotoxic activity against S100-β–loaded targets was observed.

Several differences between the present study and those using other peptide antigens to prevent T1D could explain the differences. First, GFAP-derived peptide epitopes were identified using algorithms predicting high binders, whereas our S100-β–derived peptide epitopes are true NPPEs with low or no detectable affinity for HLA-A2.1 using an HLA stabilization assay. There is a correlation between class I binding affinity and immunogenicity of CTL peptide epitopes (42). It is believed that peptide epitopes targeted by CTLs administered systemically in high doses and in aqueous solutions induce CTL peripheral tolerance, probably due to the deletion of peptide-specific CTLs (43, 44). For example, a single subcutaneous vaccination using a peptide derived from the adenovirus type 5 early region 1A oncogene induce a specific CTL tolerization against adenovirus type 5 early region 1A oncogene–expressing tumor cells; however, vaccination with a different peptide (human papillomavirus type 16 E7_49__–57_) in similar conditions induces protection in mice challenged with human papillomavirus type 16–transformed tumor cells (44). An important difference between both peptides is their affinity for the restriction element (H-2D^b^), with a higher binding affinity showing for the first peptide. For class II–restricted peptide epitopes, peptides with higher MHC binding affinity showed a higher capacity to prevent experimental autoimmune encephalomyelitis and induce tolerance when administered in conditions similar to those shown in the present study (45).

Second, MHC class I–restricted GFAP peptides (21) were administered in incomplete Freund’s adjuvant (IFA) compared with saline in the present study, and this may contribute to their *in vivo* stability. In cancer animal models, peptide/IFA vaccination primed tumor-specific CD8^+^ T lymphocytes. Those cells accumulated and persisted at the antigen-rich vaccination site, where they became dysfunctional and underwent apoptosis, resulting in hyporesponsiveness to subsequent peptide boosts. Vaccination using the same peptides in saline shifted T-cell location toward tumors, inducing anti-tumor activity and reducing systemic T-cell dysfunction (46). These data suggest that antigen clearance promotes CD8^+^ T-cell survival and generation of memory T cells, whereas chronic antigen stimulation leads to tolerance (47). Performing experiments with S100-β–derived NPPEs in IFA will help to test this hypothesis.

Multiple immunizations using an islet-specific glucose-6-phosphatase–related protein–derived peptide in saline protects from diabetes development (30). Repeated triggering of high-avidity CD8^+^ T cells with a low-avidity, but not with a high-affinity, peptide in the periphery before development of complete insulitis induces tolerance, even when peptides tested show a high affinity for the K^d^ MHC molecule. This protection is mediated by the elimination of high-avidity CD8 cells and their substitution by low-affinity peptide-specific cells. There are no data regarding the frequency or avidity of S100-β–specific CD8^+^ T cells in the A2.1-transgenic NOD mice; hence, we speculate that a combination of low affinity for MHC molecules and a quick peptide clearance from the injection site leads to the expansion of high-avidity CD8^+^ T cells, explaining the acceleration of T1D in vaccinated animals and the simultaneous increase in cytotoxicity against targets preincubated with S100-β NPPEs.

Conflicting results regarding a successful outcome (*i.e.*, diabetes prevention) has also been shown for class II–restricted peptide epitopes recognized by CD4^+^ T lymphocytes. The use of the insulin peptide epitope B9:23 (SHLVEALYLYCGERG) in immunotherapy in the NOD mice has led to 50% protection (48), no effect (49), or an increase in disease incidence (50). To explain these discrepancies, it has been proposed that efficient regulatory T cell induction needs individual peptide optimization and is greatly influenced by the activated status of the target T cells.

Finally, although both S100_10-18_ and S100_20-28_ are NPPEs eluted from HLA-A2.1 molecules, present results show that A2.1-negative T1D patients also respond against these peptide epitopes. These results are not unexpected since previous results have shown a great majority of peptide epitopes elicited responses by individuals not expressing the original restricting HLA molecule, due to promiscuous presentation via two or more HLA class I molecules (51).

In summary, we have identified NPPEs derived from the calcium-binding S100-β antigen and presented by the HLA-A*02:01 molecule. Those CTL peptide epitopes are recognized more frequently by lymphocytes from both ND and long-term T1D patients compared with HDs. Similar responses against these NPPEs can be detected in A2.1-transgenic NOD mice as early as 4 wk of age. Vaccination of A2.1-transgenic NOD mice with these NPPEs seems to accelerate T1D development, increasing insulitis and triggering the expansion of S100-β–specific CTLs, probably due to the expansion of high-affinity S100-β–specific cytotoxic T cells, suggesting that the use of short HLA class I–restricted peptides to induce tolerance needs a careful evaluation of the target cytotoxic population to avoid its stimulation.

## Supplementary Material

This article includes supplemental data. Please visit *http://www.fasebj.org* to obtain this information.

## Abbreviations

A2.1: A*02:01
BB7.2: anti-human HLA-A*02:01–FITC mAb
CTL: cytotoxic T lymphocyte
ELISPOT: enzyme-linked immunospot
GAD_65_: glutamic acid decarboxylase 65
GFAP: glial fibrillary acidic protein
HD: healthy donor
HLA: human leukocyte antigen
HLA-DR4: HLA-DRB1*04:01
IA-2: insulinoma-associated protein 2
IFA: incomplete Freund’s adjuvant
K562/A2.1: HLA-A*02:01–expressing K562 cell line
LS: long standing
MHC: major histocompatibility complex
MS: mass spectrometry
ND: newly diagnosed
NOD: nonobese diabetic
NPPE: naturally processed and presented epitope
PBMC: peripheral blood mononuclear cell
pSC: peri-insular Schwann
ROC: receiver-operating characteristic
SI: stimulation index
TBS: Tris-buffered saline
