# Supplementary material for: Naturally presented HLA class I–restricted epitopes from the neurotrophic factor S100-β are targets of the autoimmune response in type 1 diabetes
Source: FASEB J. 2019 Feb 28;33(5):6390–401. doi: 10.1096/fj.201802270R (PMC6463915; doi:10.1096/fj.201802270R)
Supplement: Supplementary file 1 [file fj.201802270R.sf1.pdf]

Supplementary Information

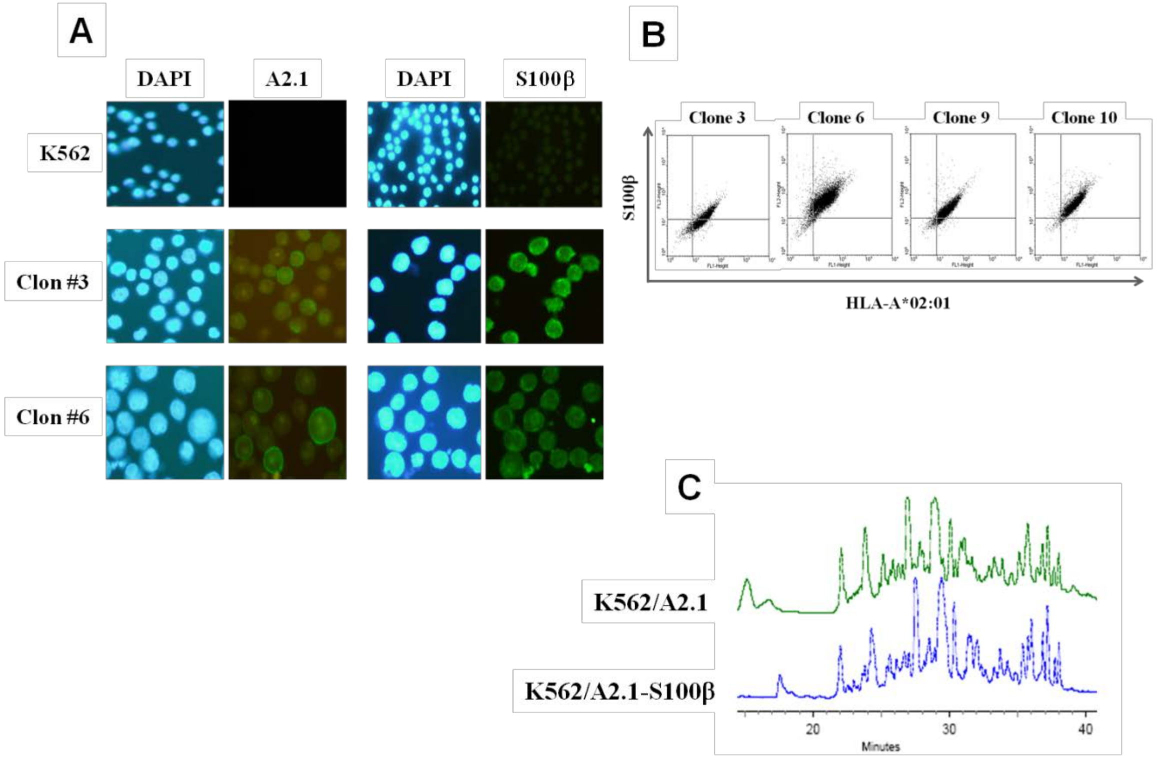

Supplementary Figure 1. Characterization of surrogate APCs and RP-HPLC fractionation of surface A2.1-bound peptides. (A) Clone 3 (Clone #3, middle panels) and clone 6 (Clone #6, bottom panels) derived from A2.1-S100β-double transfected K562 cells were stained for surface A2.1 (A2.1 columns) and intracellular S100β (S100β columns) and analyzed by immunofluorescence microscopy. Non-transfected K562 cells are shown for comparison (K562, top panels). Cell nuclei were stained with DAPI (DAPI columns). **(B)** Flow cytometry analysis of four clones of A2.1-S100β-double transfected K562 cells. **(C)** RP-HPLC chromatograms of surface A2.1-bound peptides eluted from K562/A2.1 (green) or K562/A2.1-S100β (blue) surrogate APCs.

A

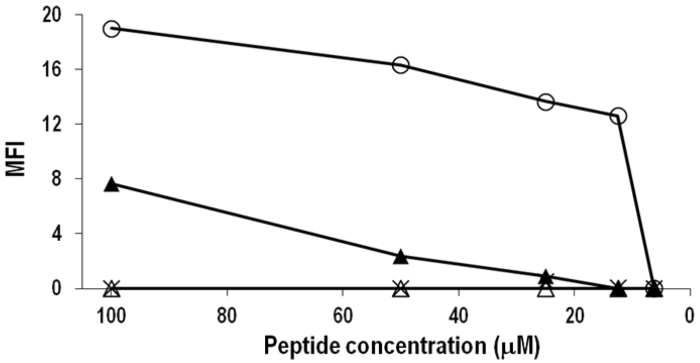

B

Predictions for HLA-A*02:01 binding affinity of S100$_{10\text{-}18}$ and S100$_{20\text{-}28}$.*

	S100$_{10\text{-}18}$			S100$_{20\text{-}28}$		
**Algorithm**	**Score (maximum)**	**Peptide score**	**Position (peptide/total)**	**Score (maximum)**	**Peptide score**	**Position (peptide/total)**
**SYFPEITHI**	22	16	8/101	22	15	12/101
**BIMAS**	28.77	0.58	14/100	28.77	0.00	70/100
**NetMHC 4.0**	99.31	3895.9	9/101	99.31	42,297.9	86/101
**RankPep**	87	60	6/101	87	2.0	44/101
**IEDB binding****	2.0	12	9/84	2.0	74	74/84
**IEDB combined*****	-0.97	-0.97	1/101	-0.97	-3.35	34/101

Supplementary Figure 2. HLA-A*02:01 binding affinity of potential S100β peptide epitopes. (A) HLA-A*02:01 peptide binding affinity was determined by the HLA stabilization assay using T2 cells. As a positive and negative controls the Influenza A Matrix 1 58-66 GILGFVFTL (empty circles) and GAD$_{65}$ 261-269 EVKEKGMAA (crosses) peptides were used. For clarity, only binding data for S100$_{10\text{-}18}$ (filled triangles) and S100$_{20\text{-}28}$ (empty triangles) are shown. No significant binding affinities could be detected for the other three S100β peptides shown in Table 1. Results are representative of three repetitions. Mean fluorescence intensity (MFI) after subtraction of background staining (MFI from untouched T2 cells) is represented in y-axis. **(B)** HLA-A*02:01 binding predictions for S100$_{10\text{-}18}$ and S100$_{20\text{-}28}$ by different algorithms for 9-mer peptides. *Score (maximum): maximum value given for all 9-mers from the S100β amino acid sequence. Peptide score: value for S100$_{10\text{-}18}$ or S100$_{20\text{-}28}$. Position (peptide/total): position of S100$_{10\text{-}18}$ or S100$_{20\text{-}28}$ / all peptides predicted by the algorithm. **SYFPEITHI:** numerical value. **BIMAS:** Half-time dissociation time. **NetMHC 4.0:** Affinity (nM). **Rankpep:** numerical value. ****IEDB binding:** prediction for HLA binding as a percentile rank. *****IEDB combined:** numerical value combining predictions for HLA binding, processing by the proteasome and TAP binding.

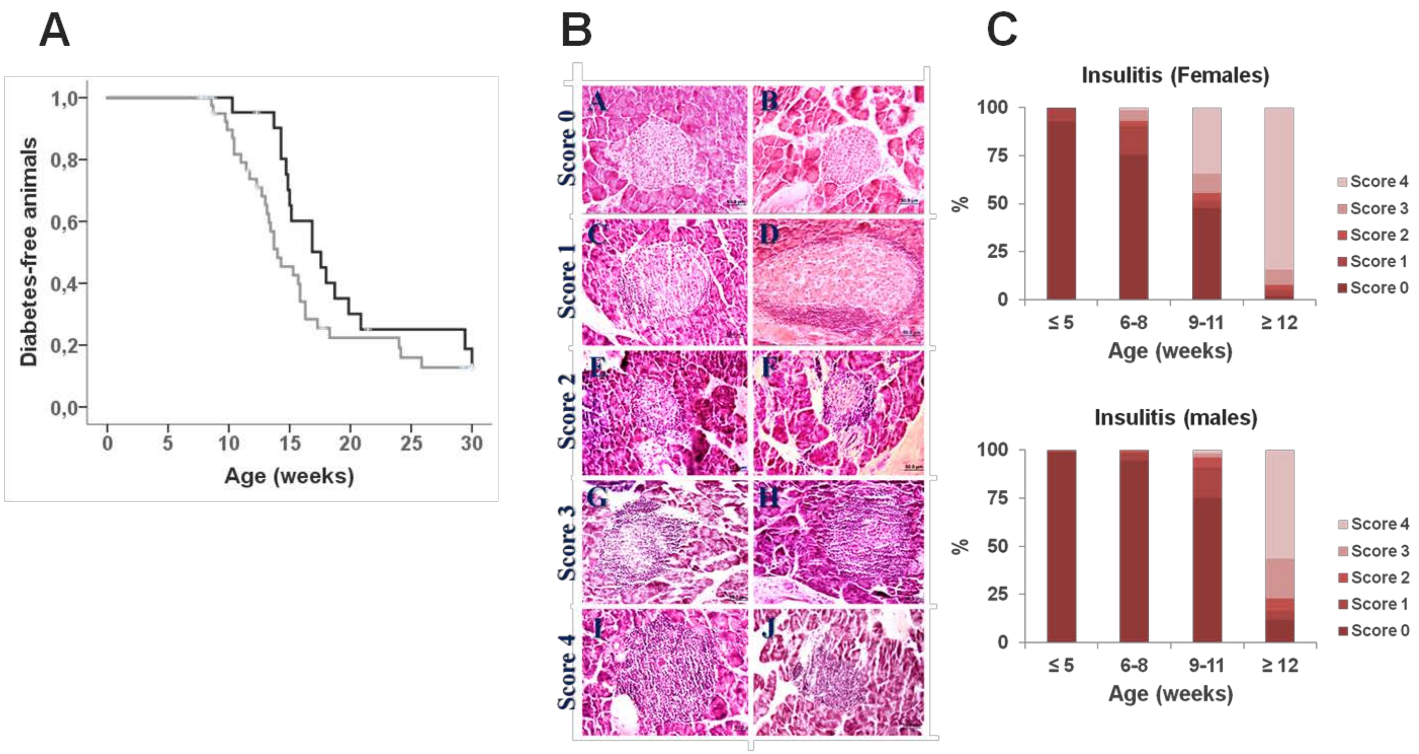

Supplementary Figure 3. Characteristics of the A2.1-transgenic NOD mice colony. (A) Mice (males=22; females=42) were monitored weekly for the development of diabetes until 30 weeks of age. Female mice (grey) develop T1D faster compared to male (black) (p=0.048; Kaplan-Meier, Tarone-Ware). **(B)** Insulitis scoring was done according to the following scale: score 0 (no insulitis, a-b), score 1 (peri-insulitis, c-d), score 2 (intra-insulitis ≤ 20%, e-f), score 3 (partial insulitis ≤ 50%, g-h), score 4 (insulitis, i-j) using hematoxilin-eosin staining of pancreas sections fixed in Bouin. Two representative images for each score is shown. **(C)** When animals reach the desired age, they were sacrificed to obtain pancreas for insulitis scoring. 5-6 animals per age group were used and at least 50 islets were scored per pancreas. Female mice (top panel) develop insulitis earlier and faster compared to male mice (bottom panel). The percentage of islets highly infiltrated (scores 3+4) are significantly higher in females compared to males from week 6 onwards (p=0.0001, Two-Tailed Fisher´s Exact Test).

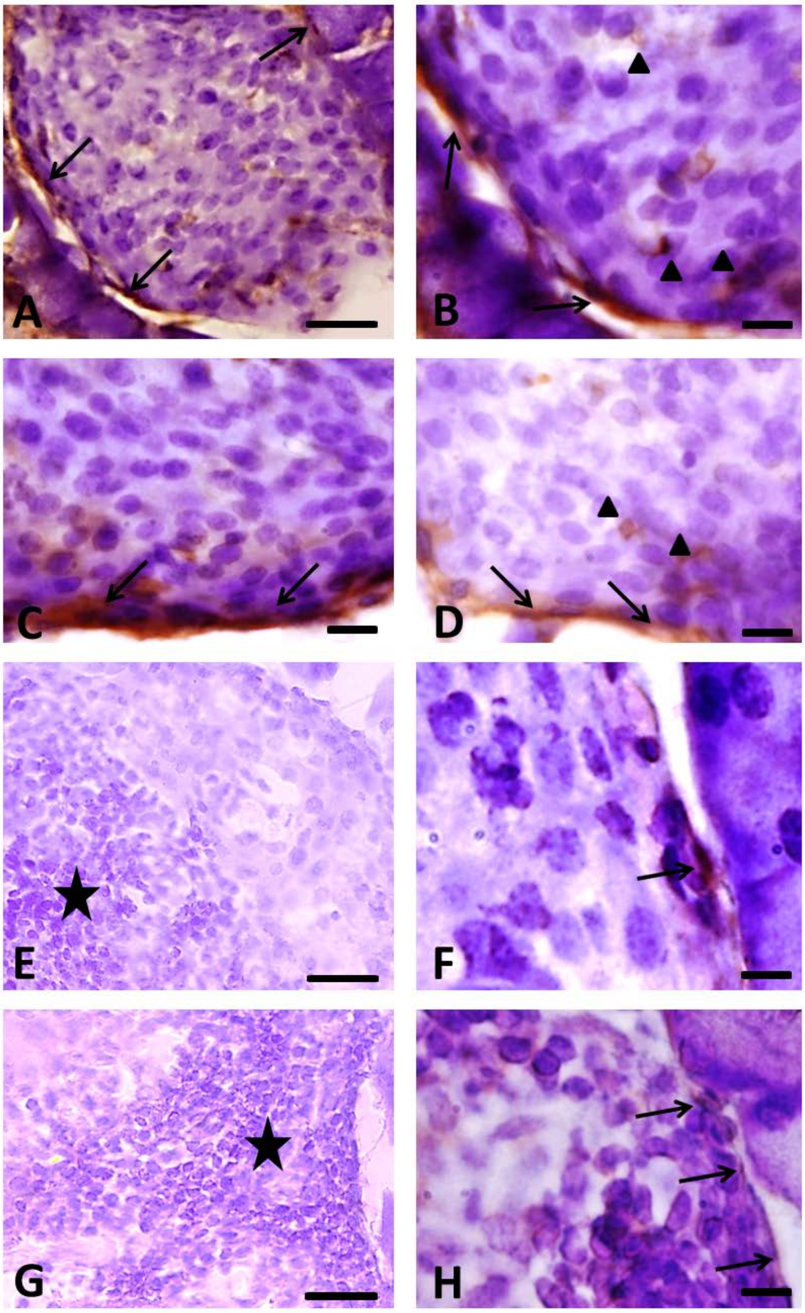

Supplementary Figure 4. Anti-GFAP immunostaining of A2.1-transgenic NOD pancreas from S100β-immunized mice. Photomicrographs from pancreases obtained from control female mice (4-weeks of age) **(A-D)** and S100β-immunized diabetic female mice with a high degree of insulitis **(E-H),** were immunostained with an anti-GFAP monoclonal antibody and counterstained with hematoxylin-eosin. Representative images are shown at higher **(B, C, D, F, H)** and lower **(A, E, G) magnification**. Numerous GFAP-immunoreactive cells (brown, arrows) are observed around the pancreatic islets of control animals **(A-D)** in contrast with the practically inexistent immunoreactivity observed in S100β-immunized mice **(E-G)**. Arrowheads: intraislet GFAP positive cells. Stars: insulitis. Scale bars: 50 µm (A, E, G); 10 µm (B, C, D, F, H).
